# Conceptual Models of Food Choice: Influential Factors Related to Foods, Individual Differences, and Society

**DOI:** 10.3390/foods9121898

**Published:** 2020-12-18

**Authors:** Pin-Jane Chen, Marta Antonelli

**Affiliations:** 1 Barilla Center for Food & Nutrition Foundation, Via Madre Teresa di Calcutta, 3/a, 43121 Parma, Italy; marta.antonelli@external.barillacfn.com; 2 Division on Impacts on Agriculture, Forests and Ecosystem Services (IAFES), CMCC Foundation—Euro-Mediterranean Centre on Climate Change, Viale Trieste 127, 01100 Viterbo, Italy

**Keywords:** consumer behavior, context of choice, healthy and sustainable food choices, decision making, food system

## Abstract

Understanding individual food choices is critical for transforming the current food system to ensure healthiness of people and sustainability of the planet. Throughout the years, researchers from different fields have proposed conceptual models addressing factors influencing the food choice, recognized as a key leverage to improve planetary and human health. However, a multidisciplinary approach is needed to better understand how different factors are involved and interact with each other in the decision-making process. The present paper reviews and analyzes existing models, providing an intact point-of-view by integrating key elements into a bigger framework. Key determinants of general food choice are identified and categorized, including food-internal factor (sensory and perceptual features), food-external factors (information, social environment, physical environment), personal-state factors (biological features and physiological needs, psychological components, habits and experiences), cognitive factors (knowledge and skills, attitude, liking and preference, anticipated consequences, and personal identity), as well as sociocultural factors (culture, economic variables, political elements). Moreover, possible directions of influence among the factors towards final food choice were discussed. The need of multidisciplinary impulses across research field with the support of empirical data are crucial for understanding factors influencing food choice as well as for enriching existing conceptual models. The framework proposed here would serve as a roadmap for facilitating communications and collaborations between research fields in a structural and systematic way.

## 1. Introduction

### 1.1. Consumer Food Choice: An Important Role in Achieving Healthy and Sustainable Food System

Global food systems possess a complex and multi-faceted set of challenges, regarding both human and environmental health, from farm to fork. From a human society point of view, there are still 690 million people who suffer from hunger while food insecurity is predicted to increase due to the present Coronavirus disease 2019 (Covid-19) pandemic and the consequent economic shock [1]. Meanwhile, 677.6 million adults, equal to 13.1% of the population worldwide, are obese [2], resulting in a double burden of malnutrition. The high prevalence of overweight and obesity, especially in urban areas, can be related to a change in lifestyle, low levels of physical activity [3], and unhealthy diets [4] based on the interaction between individual characteristics on food choices and obesogenic environments. Especially, obesogenic environments are recognized as crucial drivers of the increasing prevalence of obesity epidemic [5,6], including microenvironments at individual level (e.g., school, workplace, home, neighborhood) and macroenvironments at societal level (e.g., education and health systems, government policy, society’s attitudes and beliefs) [7]. The outcome of food choice is based on interactions between environmental and individual factors [8].

Individual food choices, embedded in the pattern of food consumptions, evolved according to the changes of natural environment, biological basis, physical need, lifestyle, and development of technology [9]. In the modern society, owing to increasing national wealth and urbanized living, people consume more animal proteins as well as processed food. At the same time, consumptions of whole food or minimally-processed foods such as whole grains, legumes, and other sources of fiber decreased [10]. Some studies have highlighted that eating patterns and food choice have changed with the change of global food systems and food supply, resulting in a shift toward increased intake of unhealthy food [11]. The change of global food supply chains influences the food environments, [12]. Particularly, food choice with ultra-processed food significantly increased, owing to the easy access, cheap price and marketing strategies [13,14,15]. The vicious cycle has been created between food choice and the consequence of food choice as it is confirmed that consumption of heavily-processed foods is significantly associated with higher BMI and increased likelihood of being obese [13,14,16]. On the other hand, overweight and obese individuals tend to show more liking and to select more energy-dense foods [17,18,19].

Given that negative impacts on human beings as well as on the planet (e.g., pre- and post- production activities in food system produces up to 37% of the total anthropogenic Greenhouse Gas emissions [20]) have considerably grown, promoting healthier and more sustainable food choices and better diets have been a new multidisciplinary research impulse [21,22,23,24,25]. According to Food and Agriculture Organization [26], sustainable healthy diets are dietary patterns that promote all dimensions of individuals’ health and wellbeing; have low environmental pressure and impact; are accessible, affordable, safe, and equitable; and are culturally acceptable. Grunert [22] has pointed out that consumers have great potential in making food chains more sustainable by choosing more sustainable food production and rejecting less sustainable alternatives through their food choices. For example, choosing low-impact foods (e.g., minimally-processed plant-based foods) and increasing use efficiency of agricultural input offer larger environmental benefits [27]. While food choices with heavily-processed food have negative impacts on the environment [28], lowering consumption of more discretionary products (e.g., oils and sugar) can reduce land use, emission, and freshwater withdrawals [29]. Thus, promoting healthier and more sustainable dietary patterns, rooted in food choices at individual level, has been recognized as a potential and crucial solution [30]. A number of multidisciplinary studies have shed light on the importance of addressing the role of individual food choice in tackling the current nutrition and also environmental crisis [31,32,33].

### 1.2. Exploring Factors Influencing Consumer Food Choice and Constructing the Conceptual Models

#### 1.2.1. Three Main Categories of Factors Influencing Food Choices: Food-Related Features, Individual Differences, and Society-Related Features 

A rich body of literature has focused on exploring factors influencing individual food choice. Due to the complex nature of food choice, proposed factors as well as the categorization of factors differed from one study to another. However, although works from different research fields (e.g., nutrition, psychology, social science, marketing, etc.) provided evidence with different perspectives, the factors affecting food choices can be leveled into three main categories: (1) Food-related features: intrinsic features such as color and aroma, and extrinsic features such as information and packaging [34,35]); (2) individual difference: biological (e.g., hunger, appetite, and taste), physical (e.g., access, skills of cooking, and time), psychological (e.g., mood and stress), cognitive (e.g., attitudes or preference, beliefs, and knowledge), and social (e.g., family, and peers) factors (see [36,37,38]); (3) society-related features: culture, economic variables such as price and income, and policy, e.g., [39].

#### 1.2.2. The Role of Food Environments as Factors Influencing Food Choices

In addition to the three main categories, in recent years, ‘food environments’ have been defined and recognized as important factors influencing people’s food choice. According to Swinburn, et al. [40], food environment equals to the collective physical, economic, policy, and sociocultural surroundings, opportunities, and conditions that influence people’s food choices and nutritional status. In fact, ‘food environments’ include different factors from the aforementioned three main categories, such as physical and social environments as well as economic, policy, and sociocultural environments. Some studies attempted to provide a more holistic point of view by integrating the role of food environments. For example, an early paper [41] proposed that food consumption is based on food preference, under the influence of food characteristics (e.g., taste, texture, and cost), individual characteristics (e.g., nutritional status, knowledge, and attitudes to health), and environment characteristics (e.g., season, degree of urbanization, and size of family). Rozin [42] specified the influence of biological (physiological and evolutionary/adaptive), psychological (preference and context), social (sociology), and cultural (anthropology) factors on food choice. 

It has been concluded that there are social and environmental influences on food choice (e.g., modelling influences, eating competence family environment, food labels, taste, appearance, personal food history, habits, and familiarity) as well as psychological influences on eating behavior (perceived behavioral control and motivation) [43]. Leng, et al. [44] pointed out the determinant of food choice, including dietary components (e.g., highly palatable foods), physiological mechanisms (e.g., neural mechanism of hunger and satiety as well as motivation and reward based on foods), cognitive-affective factors (perceived stress, health attitude, anxiety, and depression), familial, genetic, and epigenetic influences on personality characteristics, and diverse cultural and social pressures. Castro, et al. [45] focused on factors influencing choice in food retail environments such as shelf display and product factors (shelf display, branding, nutrition labeling, and food sampling), pricing and price promotion factors, in-store and customer decision-making factors (customers’ implicit beliefs about the relationship between taste and healthfulness), and store environment factors (e.g., smaller aisles). Bauer and Reisch [46] summarized that food decisions are affected by individual (psychological, physical, neurological), social, and environmental factors. 

#### 1.2.3. Development of Early Conceptual Models of Food Choice as the Prototypes

It is recognized that food choices are multifaceted, situational, dynamic, and complex [47]. Thus, a multidisciplinary approach and a holistic picture are needed to understand not only how different factors are involved but also how the factors are structured and interact with each other in the decision-making process. To this aim, comprehensive conceptual models of food choice behavior have been developed for understanding the process of making food choices. Furst, et al. [48] proposed the model with factors involved in food choice being categorized into three components: life course, influences, and personal system. According to the authors, the life course includes the personal roles and the social, cultural and physical environments to which a person has been and is exposed. A person’s life course generates a set of influences: ideals, personal factors, resources, social framework and food context. These influences inform and shape people’s personal systems, including conscious values, negotiations and unconsciously operationalized strategies that may occur in a food-related choice situation. In another model [49], features of food, personal state, and socio-economic factors were included. The features of food (e.g., chemical properties and nutrient content) can trigger physiological effects (e.g., hunger) that directly influence food choice. Moreover, food features can influence a person’s perception (e.g., taste and texture) which contribute to the formation of attitudes under the influence of socio-economic context (e.g., price, brand, and culture). The attitudes then influence the output of food choice. Finally, personal psychological factors such as personality, mood, and beliefs can influence final food choice directly or by affecting the attitudes. Similarly, the model proposed by Steenkamp [50] demonstrated that properties of food (physiological effects and sensory perception) personal-related factors (biological, psychological, socio-demographic), and environmental factors (economic, cultural, marketing) all contribute to the food decision process which involves need recognition, search for information, evaluation, and the final food choice.

In addition, Grunert, et al. [51]’s total food quality model distinguishes “before” from “after” purchase evaluations. Cost cues, extrinsic quality cues, intrinsic quality cues, and the perception of these cues all contribute to expected quality (taste, health, convenience, and process), which influence purchase motive fulfillment and intention to buy. Moreover, this model includes the time domain, showing the important influence of experienced quality after purchase on future choices. So far, all these models focus on individual and social determinants of food choice. Sobal, et al. [52] proposed that factors in the bio-physical environment (e.g., biodiversity, land, air, water, energy) as well as in the social environment (e.g., knowledge, capitals, policy) affect the consumer behavior.

These conceptual models of food choice can be seen as the prototype. They introduced not only factors involved in the food decision-making process but also constructed the model emphasizing the relationship among the factors and indicating the process or pathway contributing to the final food choice.

### 1.3. Aim of This Review and the Proposed Framework

Individual food choice is crucial as it largely affects our health and our planet, with multifactorial determinants rooted in food-related features, individual differences, and society-related features. Moreover, interactions between factors also contribute to the final food choices via direct and/or indirect mechanisms. It is important to understand factors influencing our food choice and thus possible interventions and policy recommendations can be applied for improving food choice to successfully transform the food systems. In addition to early conceptual models of food choice, in recent years, there is abundant literature focusing on expanding and enriching the conceptual models of food choice. However, no single perspective, theory, framework, or model can provide an entire picture of food choice mechanism and properly explain it as influential factors have been categorized in different ways. For example, even though most of the models considered physiological factor as individual difference, some models referred it as a factor of properties of food e.g., [50]. Factors influencing food choice are not clearly leveled across domains of food itself, individuals, and society. Thus, the present paper aims to (1) systematically review existing conceptual models of food choice; (2) summarize and re-categorize factors affecting food choices following the three main categories: food-related features, individual differences, and society-related features; (3) analyze the direction of influences among factors in the conceptual models; and (4) develop and provide a conceptual framework which disentangles the complex and multifactorial nature of individual food choice. Our framework is developed from Eertmans, Baeyens and Van Den Bergh [34]’s model with the categorization of factors influencing food choices as follows: food-internal factors, food-external factors, personal-state factors, cognitive factors, and sociocultural factors.

## 2. Materials and Methods 

**Figure 1:**
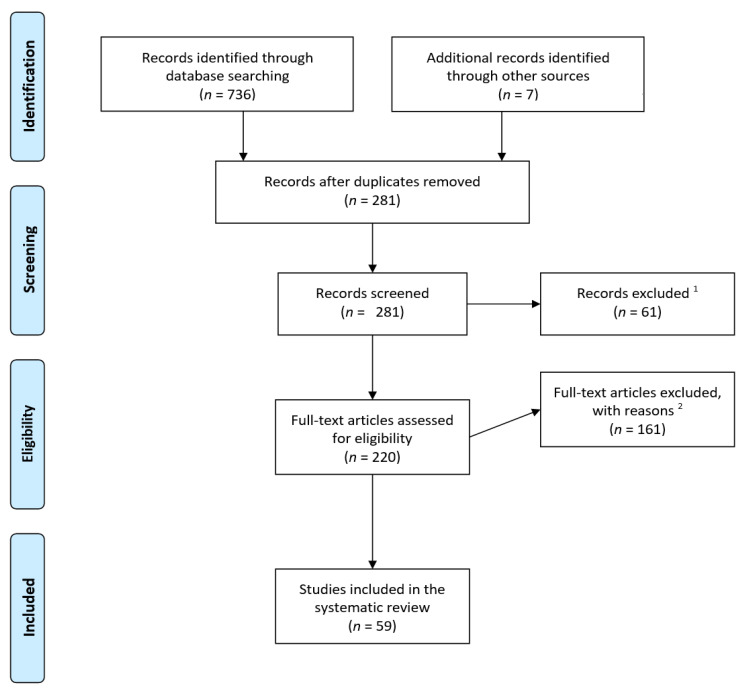
PRISMA flow diagram indicates the selection process of publications related to conceptual models of food choice: ^1^ 61 records were excluded (1 non-English publication, 1 comment, 59 unrelated to the topic of factors influencing consumer food choice); ^2^ 32 publications discussing factors affecting food choice without proposed conceptual models were excluded; 18 publications reported non-conceptual models (e.g., economic-psychological model, computation models, predictive models, etc.) were not included; 21 publications focusing on factors affecting other food-choice-related dependent variables (e.g., willingness to pay, nutritional label use, choice of brand, etc.) were rejected; 11 publications targeting non-healthy-adult population (preschoolers, adolescents, order adults, people with eating disorder, etc.) and 31 publications addressing food choice within specific sociocultural context (e.g., Brazilian Amazon, two urban food deserts, low-income consumers, etc.) were excluded. We excluded also 16 publications addressing food choice of specific food (e.g., traditional food, functional food, snacks, etc.) and 22 publications emphasizing the intervention for improving food choice and 10 publications examined the methodology or tools (e.g., questionnaire and interview) for measuring food choice were not included. A final set of 59 publications has been analyzed.

The present review aims at introducing factors influencing individual food choice with a proposed conceptual model by including academic publications as well as gray literature. The inclusion of publications is based on the following criteria: (1) studies had to be published in English; (2) studies which were based on healthy adult population; (3) studies focused on general food choice instead of specific food choice (e.g., ethnic food and functional food); (4) studies which were conducted not within specific social cultural context (e.g., specific to certain community or town); (5) studies which proposed at least a conceptual model of food choice. The following databases were used for our search: PubMed, Science Direct, and Google Scholar with keywords as follows: ‘Consumer food choice factor’ and ‘Food choice conceptual model’. The selection process followed the PRISMA guideline (Figure 1). In total, 280 records were screened, and 61 records were excluded (1 non-English publication, 1 comment, 59 unrelated to the topic of factors influencing consumer food choice). For full-text articles assessed for eligibility, first, 32 publications discussing factors affecting food choice without proposed conceptual models were excluded. A total of 18 publications reported non-conceptual models (e.g., economic-psychological model, computation models, predictive models, etc.) were not included. A total of 21 publication focusing on factors affecting other food-choice-related dependent variables (e.g., willingness to pay, nutritional label use, choice of brand, etc.) were rejected. Second, 11 publications targeting non-healthy-adult population (preschoolers, adolescents, order adults, people with eating disorder, etc.) and 31 publications addressing food choice within specific sociocultural context (e.g., Brazilian Amazon, two urban food deserts, low-income consumers, etc.) were excluded. We excluded also 16 publications addressing food choice of specific food (e.g., traditional food, functional food, snacks, etc.). Finally, 22 publications emphasizing the intervention for improving food choice and 10 publications examined the methodology or tools (e.g., questionnaire and interview) for measuring food choice were not included. A final set of 59 publications has been analyzed. The analysis focuses on two outcomes. First, we summarized the factors influencing food choice including intrinsic and extrinsic features related to food, individual differences in personal state and in cognitive functions, and factors at societal level such as culture, economy, and policy. Second, we introduced the structures of conceptual models of food choice and indicated the direction and interaction of aforementioned factors in the decision-making process. Details on papers included in the review are reported in Supplementary Materials Table S1.

## 3. Results

**Figure 2:**
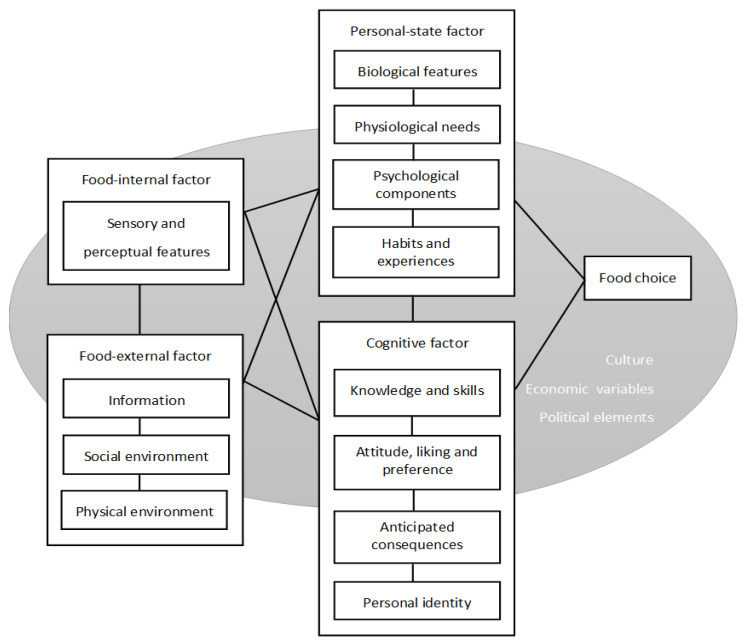
The proposed framework of factors influencing food choice developed from Eertmans et al. (2001)’s model [34]. The lines in the figure indicate the interactions between different factors.

Each conceptual model of food choice was analyzed with factors included in the models being categorized into the following main factors affecting food choice according to our proposed framework developed from Eertmans, Baeyens and Van Den Bergh [34]’s model (Figure 2): food-internal factors, food-external factors, personal-state factors, cognitive factors, and sociocultural factors. Table 1 lists in detail all the factors included in these main factors. We also summarized the directions of influence among the factors from available models.

### 3.1. Factors Influencing Food Choice in Conceptual Models

In this section, we summarize the basic factors included in the conceptual models of general food choices.

#### 3.1.1. Food-Internal Factor: Sensory and Perceptual Features

Food-internal factor is defined as features possessed by the food itself such as sensory (e.g., flavor, taste, smell, and texture) and perceptual (e.g., color, portion size, nutrition and health value, and quality) properties. Twenty-six models have proposed that the sensory and perceptual features influence the food choice [21,32,34,41,49,50,51,53,54,55,56,57,58,59,60,61,62,63,64,65,66,67,68,69,70,71]. A review paper summarized that visual and odor cues contribute to identifying food in the near environment, guiding food choice and memory for eating, while tastes and textures influence meal size and the development of satiety after consumption [72]. Another literature review concluded that odor exposure induces appetite while taste and texture contribute to satiation based on eating rate and oral exposure duration of food in the mouth, playing an important role in a (macro)nutrient sensing system [73].

#### 3.1.2. Food-External Factors: Information, Social Environment, Physical Environment

In our definition, information about the food item (e.g., nutritional labels, health claims, packaging, aesthetics, ethics of production history, brand, and advertisement) is defined as one of the food-external factors. Twenty-eight models have included food-related information as factors influencing the food choice [21,22,24,32,34,49,50,51,53,55,56,57,58,61,62,64,65,70,74,75,76,77,78,79,80,81,82,83]. In addition to these models, there is a rich body of literature focusing on the effects of food label and food label use on food choice. Food labeling provides information on essential characteristics of food items and food label use has been recognized as an important component of strategies tackling unhealthy diets and obesity. For example, Cowburn and Stockley [84] reviewed papers reporting consumer understanding or use of nutrition labels and concluded that improvements in presenting and designing nutrition labeling could make a contribution towards making the existing point-of-purchase environment more conducive to the selection of healthy choices. Interpretational aid such as verbal descriptors and recommended reference values is needed to help consumers assess the nutrient information in order to improve overall diet. Cecchini and Warin [85] did a meta-analysis and confirmed that nutrition labeling, especially interpretive labels (e.g., traffic light labels), may be an effective approach to empowering consumers in choosing healthier products and in reducing calorie intake. More recently, a review explored how consumers value and response to nutrition information on food labels against information on environmental and/or social responsibility [86]. Consumers generally have a positive view of environmental and social responsibility food labeling schemes while the most preferred attribute was organic labeling, inferring to information related to health. In brief, interpretive nutrition labels with the combination labeling containing healthy and sustainable attributes can be propriate and effective in promoting healthy and sustainable food choice. Oostenbach, Slits, Robinson and Sacks [57] examined the effect of nutrition claims on food choice. The influences of nutrition claims depend on the type of claim and food with the claim. For example, nutrition claims can influence consumers’ perception that the product is healthier and less tasty. However, nutrition claims can make the appropriate portion size appear larger, resulting in the underestimation of the energy content of food products. Nutrition claims also influence food purchase intentions, moderated by the perceived healthfulness of the relevant food products and the health consciousness of individuals.

In addition to food-related information, the food environments are considered as food-external factors which also largely affect the food choice. We further divided food environments into two factors, namely social environment (e.g., intrapersonal factor and social norms from family, peers, and media including ethical concern, as well as social context when food choice is made) and physical environment (e.g., product availability, accessibility, and convenience; in-store characteristics such as shelf display, order, placement, and time). Thirty-four models indicated that social environment contributes to the food choice decision [21,23,34,41,47,48,52,53,55,56,59,60,62,70,71,75,76,77,78,82,83,87,88,89,90,91,92,93,94,95,96,97,98,99]. In the social environment, family and the home food environment are important influences on dietary intake but this influence is more profound for children and adolescents, not adults [100]. Instead, for adults, individual food choices are influenced by interactions with others beyond the family unit (e.g., coworkers, peers, and close friends). It is suggested that not only the context of shared meals but also social norms and attitudes among members of a group have impacts on types or amounts of foods that individuals consume no matter whether people eat together or not [100]. Indeed, social modeling determines what and how much people choose to eat, using others’ eating behavior as a guide. The effect increases when individuals desire to affiliate with the model or perceive themselves to be similar to the model. The effect reduces for choosing specific food (e.g., healthy food or snack foods) as well as meals (e.g., breakfast and lunch) [101].

Twenty-eight models pointed out that physical environment contributes to the food choice decision [23,32,34,41,48,49,51,52,53,55,58,59,61,62,66,70,71,75,76,80,82,87,88,89,90,92,94,102]. For the physical environment, studies have indicated that food choices and eating behaviors, as well as risk for obesity, are influenced by the physical availability and accessibility of food products in the workplace, surrounding neighborhood, retail food stores, as well as restaurants [100]. A review done by Castro, Majmundar, Williams and Baquero [45] confirmed that food retail environments do shape customer food choices of healthy or unhealthy items and energy intake, including product location on the shelf, the appearance of the products on the shelf, the brands available (i.e., customers are more likely to choose familiar over unfamiliar brands), as well as product attributes. For example, feeling confined by smaller aisles may encourage consumers to make more varied choices [45]. Finally, some models also included ‘time’ as an influential factor, mainly referring to time availability for food preparation and home cooking [48,59,70,75,92]. According to a review, lack of time can result in changes in food consumption patterns such as a decrease in food preparation at home, and an increase in the consumption of convenience or ready-prepared foods [103].

#### 3.1.3. Personal-State Factors: Biological Features and Physiological Needs, Psychological Components, Habits, and Experiences

In our definition, personal states which affect food choice include biological features (e.g., genetic factors, personal dietary patterns and metabolism, physical condition such as health) and physiological needs (e.g., hunger, appetite, and weight status), psychological components (e.g., emotion, motivation, personality), and personal habits and experiences. Sixteen models have included biological features and physiological needs as factors affecting food choice [21,48,49,50,53,55,57,61,66,71,77,83,88,89,90,95]. Except for biological features which are difficult to change, dietary decisions can be regulated by physiological functions such as circulating metabolic hormones and neural mechanism involved in food intake and appetitive behaviors [44]. Vice versa, extreme and narrow food choices could result in fluctuation and unbalanced weight and health status (i.e., the strongest predictors of weight gain were dieting and unhealthy weight control behaviors) [44].

Psychological components appeared in twenty-nine models [21,23,25,34,47,48,49,50,51,52,55,56,57,59,60,62,66,71,74,79,80,83,88,89,91,93,95,98,102]. According to these models, the majority of studies focused on how emotion is involved in food decision-making. For instances, the model proposed by Gutjar, de Graaf, Kooijman, de Wijk, Nys, Ter Horst and Jager [74] was dedicated to exploring the role of food-evoked emotion in food choice. Food-evoked emotions, which can be sorted into two dimensions (valence and arousal), add predictive value to solely liking ratings, and may guide consumers’ choice behavior. For taste-based choice, the combination of liking and emotional valence had stronger prediction. For package-based choice liking, emotional valence and emotional arousal together have a stronger predictive value [74]. In addition to emotion, motivation is also frequently involved in the models. However, the motivation to choose one food over another is driven by the emotional, hedonic and metabolic properties of the foods [44]. In other words, the motivation is actually driven by other factors. Thus, we did not include “motivation” as a factor in our categories.

Comparably fewer models mentioned about previous experiences and/or habits [21,47,49,61,71,75,83,89,90]. In these models, experiences were categorized as a psychological factor related to the function of memory while habits were categorized as a situational factor. However, experiences and habits are composed by multi-factors such as emotion, memory, and learning [55]. Moreover, the process of consciousness needs to be considered [104,105]. Thus, in our opinion, when we include experiences and habits under the frame of food choice, it is better to categorize them into personal-state factor, stressing the contribution of experiences and habits to the person at the moment of making food choice.

#### 3.1.4. Cognitive Factors: Knowledge and Skills, Attitude, Liking and Preference, Anticipated Consequences, and Personal Identity (Demographic Features, Belief, and Value)

Before reaching the final output of food choice behavior, cognitive factors have their impacts on food decision-making. Possessing knowledge (especially nutritional and food-related knowledge) [24,34,48,52,56,57,58,64,70,78,79,82,88,93,94,97,106,107] as well as food management skills [41,48,52,75,83,90,92,97] can have great influence on food choice. For example, nutrition knowledge has been shown to be a partial mediator of the socio-demographic variation in food intake, especially for fruit and vegetables, implying that knowledge is an important factor in explaining variations in food choice [108]. 

The second factor is evaluation-based [50], including attitude [21,24,25,41,48,49,56,59,60,61,68,69,70,79,82,90,91,92,95,96,97,102,107,109,110,111], liking [34,63,74,88], and preference [58,69,87,94]. The potential differences among these three components towards food choice can be as follows: attitude represents the implicit evaluation towards food items. Usually, the attitude is based on valence (positive and negative) but it can be also based on other concepts (e.g., healthiness) [112]. Liking, instead, focus on the sensory evaluation of food [113]. Finally, preference is based on comparison (i.e., preferring food item A than item B). For example, the preference of obese people for foods was affected more by fat content than by carbohydrate or sucrose content [114].

The third cognitive factor includes expected consequences [34,48,51,54,63,64,66,68,76,77,78,88,92,95,109,110] regarding the concern for health consequences (e.g., benefits or risks) of food consumption [34]. For example, consumers are prone to choose foods which give desirable consequences such as expected longer life [115]. 

Last but not least, personal identity, including demographic features such as age, gender, ethnic identity, and education [23,24,41,47,50,59,61,63,64,82,87,88,89,90,91,93,94,95] can also affect food choices. For instance, food choices represent personal identity as well as the story of families, migrations, assimilation, resistance, and changes over times [116]. Possessing a healthy eating identity was significantly associated with greater fruit and vegetable intakes [117]. Moreover, personal belief and value, shaped by culture and society, also contribute to the decision of food choice [21,23,25,48,49,52,59,60,61,64,70,75,77,78,82,83,90,95,96,107,111]. Indeed, two early studies have pointed out the potential role of personal belief and value in maintaining goal-directed behavior, especially for health-related food choice [118,119].

#### 3.1.5. Sociocultural Factors: Culture, Economic Variables, and Political Elements

For previous factors, the influences are mainly at individual level. The final category, sociocultural factors, focuses on societal level. According to Larson and Story [100], the influence of macroenvironment on individual food choice includes (1) income, socioeconomic status, and price of food, reported by twenty-nine models [32,41,47,48,49,50,51,52,53,54,55,56,58,59,62,65,66,69,70,75,76,79,82,87,88,90,92,93,94]; (2) cultural norms and values are reported by seventeen models [41,48,49,50,51,53,55,56,58,70,71,76,88,89,94,97,98]; (3) agricultural and food policy and regulations, reported by nine models [52,55,58,59,76,88,89,94,98].

About income and cost of food, Larson and Story [100] specified that the monetary and time costs of buying and preparing foods (e.g., nutrient-dense foods cost more than foods that are higher in energy), especially ones with health values, are additional barriers to good nutrition for low-income groups. Indeed, higher cost of healthy choices or diets can strengthen socioeconomic disparities in diet quality [120]. Nevertheless, price elasticities for foods can be also a strategy in changing consumer demand, shifting the purchase of unhealthy food to healthier food. The example [121] showed that a 10% increase in soft drink prices should reduce consumption by 8% to 10%. Price and promotion in the food retail environment do affect consumers’ purchase intentions and choice [45]. The quantity and quality of food along the supply chain can be affected by agriculture and food polices, resulting in altered food price, directly or indirectly influencing the choice, affordability, as well as the right to nutritious food of the consumer [100]. Finally, we could not ignore the fact that food choice and eating behaviors are influenced by cultural factors, especially shared values and beliefs, which shape perceptions of food and the concept of healthiness [100]. 

### 3.2. Influences among Factors in Conceptual Models of Food Choice

Thirty-two conceptual models of food choice indicated possible directions of influence among the factors towards final food choice [41,47,48,49,50,51,52,54,56,57,60,61,62,63,64,65,66,74,76,77,78,87,90,91,92,93,95,96,97,102,109,110].

#### 3.2.1. Possible Influences among Factors in Conceptual Models of Food Choice 

For most of the conceptual models, the directions of influence among factors affecting food choice are demonstrated with theoretical basis or literature review e.g., [57,61,66,76,77,90,97]. The early-established models can be seen as the porotypes [48,49,50,51,52], allowing consecutive research to expand or enrich the models with new data or evidence. About integrating and expanding existing models, as an example, Rose, Bodor, Hutchinson and Swalm [87] integrated the factor of accessibility around the neighborhood based on an economic model of food consumption. Food cost, taste, preference, and income influenced the food choice for purchasing. The food cost can be directly influenced by in-store price or indirectly affected by the placement of food store via travel cost. In-store characteristics such as product varieties as well as shelf space and placement had impacts on promotional effect and social acceptability, which then further influenced tastes and preferences. Tastes and preferences also can be directly influence by demographic variables such as age, race-ethnicity, and education. The framework proposed by Marreiros and Ness [56] combined main features of the Engel-Blackwell-Miniard model [122] regarding consumers decision-making, and the main constructs of the total food quality model [51] developed for food products concerning mainly consumers quality evaluation. The author bridged these two models with the emphasis on the relationships between them [56].

About adopting and enriching existing models, for instance, the models shown in Connors et al. [54] and in Sobal and Bisogni [47] were both adopted from Furst et al. [48] with modifications or elaborations. The life course and experience created influences including ideals, personal factors, resources, social factors, and context. In the model of Sobal and Bisogni [47], these influences then had an impact on personal food system built upon values, situation, and strategy. The personal food system affected food behavior, which then had a feedback loop in shaping the aforementioned factors (life course experience, the created influence, and personal food system). In the model, the personal food system was composite of value, managing relationships, health, taste, cost, convenience, and strategies. However, there was no feedback loop [54].

To briefly conclude, few models have clearly indicated the direction of influence among the factors with experimental data or with mathematical modeling. There is a need of empirical data to support and to disentangle the interactions between the factors.

#### 3.2.2. Direction of Influence among Factors in Conceptual Models of Food Choice

In general, the more specific factors the model addressed, the clearer indications of directions of influence could be made, supported by experimental data. For example, in the model of Gutjar et al. (2015), both sensory (intrinsic) and packaging (extrinsic) information trigger emotional responses but liking was based on sensory information [74]. Both emotional responses and liking then contribute to the prediction of food choice. 

Structural equation modeling is frequently used to investigate the directions of influence among factors towards certain food choice. For healthy food choice including fruit and vegetables, factors such as intention, action planning, and self-efficacy, with a person’s understanding of nutrition information, can better explain healthy food choice behavior [64]. The mediating roles of food mavenism, food knowledge, food involvement, and equality-universalist values were reported in increasing vegetable intakes [93]. Moreover, market availability, interest in healthy eating, and time pressure significantly influence choice of plant-based convenience foods [92]. Intention, self-identity and past behavior were proved as direct predictors of fruit and vegetable intake [91]. For food with environmentally-sustainable attributes, personal norms regarding the use of organic food affect attitudes toward the use of organic produce in institutional settings, which is partly mediated by own purchase of organic products [96]. Choice of convenience food with environmentally-sustainable attributes is positively related to consumer food shopping habits, food-related environmental behavior, gender, income, and knowledge [102]. Another study also pointed out that likelihood of buying healthy convenience food is affected by overall liking of the meal, which is affected by liking of sensory specific product attributes like appearance, flavor, and odor [63]. 

For newly emerged food such as genetically modified (GM) food, perceived benefits and risks play a significant role in shaping behavioral intentions towards GM food [110], the attitude to GM technology being the main driver of consumers’ beliefs about risks and benefits. Public attitudes toward GM food are being formed from trust in science and in public authorities under different cultural contexts, which determines consumer’s final purchasing decisions [109]. Finally, some studies examined factors affecting ethical food choice, suggesting that ethical consumption and purchase intention have a direct influence on choice behavior with significant association between social, emotional, and epistemic values with ethical consumption intentions [62]. In addition, universalism was shown having impact on food choices with less meat or free-range meat. This impact was mediated by prevention-oriented food choice motives and motive-congruent animal friendly attitudes [60]

Supplementary MaterialsTable S2 summarizes the directions of influence of factors affecting food choices based on publications with empirical data we included.

## 4. Discussion

### 4.1. Main Findings: The Multifactorial Nature of Individual Food Choice

In recent years, research as well as large scale initiatives have been launched, substantiating the imperative for individuals and governments to improve population health by taking substantial actions in the domain of individual food choice and eating behavior [11]. Since consumers’ daily food choices have great potential in transforming towards healthier and more sustainable food systems [11,22], the first and essential step before considering interventions is understanding factors influencing individual food choice in a structural and systematic way. The present paper provides an insight into the complex and multifactorial nature of individual food choice by analyzing factors included in conceptual models. 

The early development of conceptual models of food choice can be dated back to the 1990s. There were three main types of models. The first one, which can be seen as the prototype of the models, already demonstrated three levels of factors influencing the final choice, namely food features, personal system, and environment [41,48,49]. The second type focuses on the effect of price, quality, and value [51,65]. While the third one, the model proposed by Sobal, Khan and Bisogni [52] took into consideration a broader view including relationships of the food and nutrition system to other systems such as environmental system, governmental system, health care system, cultural system, economic system, and even transportation system. The conceptual models we include in the analysis mainly follow the three-level framework of food features, personal system, and environment. However, different models might include different numbers of factors with different ways of categorization within and across these three levels. Thus, the present review analyzed existing conceptual models of food choice, summarized influential factors affecting food choice, then re-categorized and integrated the results from the literature into a proposed three-level framework of factors influencing food choice, namely food-related features, individual differences, and society-related features. The ultimate goal is to provide a clear and simple roadmap for facilitating future development of research in the field of consumer food choice and maximize the contribution from individual studies. Being on the same page, the framework may help researchers communicate the idea, compare research data, and replicate existing results with ease. In our framework, influential factors determining food choice are categorized into food-internal factor (sensory and perceptual features), food-external factors (information, social environment, physical environment), personal-state factors (biological features, physiological needs, psychological components, habits, and experiences), cognitive factors (knowledge and skills, attitude, liking and preference, anticipated consequences, and personal identity), and sociocultural factors (culture, economic variables, political elements).

In our samples, five most frequently addressed factors in the models are as follows: (1) social environment which belongs to food-external factors [21,23,34,41,47,48,52,53,55,56,57,58,59,60,62,70,71,74,75,76,77,78,82,83,87,88,89,90,91,92,93,94,95,96,97,98,99]; (2) personal-state focusing on psychological component [21,23,25,34,47,48,49,50,51,52,55,56,57,59,60,62,66,71,74,79,80,83,88,89,91,93,95,98,102]; (3) economic variables such as income, socioeconomic status, and price [21,32,41,47,48,49,50,51,52,53,54,55,56,58,59,62,65,66,69,70,75,76,79,82,87,88,90,92,93,94]; (4) food-related information which also belongs to food-external factors [21,22,24,32,34,49,50,51,53,55,56,57,58,61,62,64,65,70,74,75,76,77,78,79,80,81,82,83]; and (5) physical environment which also belongs to food-external factors [23,32,34,41,48,49,51,52,53,55,58,59,61,62,66,70,71,75,76,80,82,87,88,89,90,92,94,102].

The results reflect the facts that (1) social environment is the most addressed factor influencing food choice; (2) due to the availability of research evidence, factors such as food information, food environment, and economic variables are easier to be manipulated and measured in the experimental settings. Thus, the role of these factors in influencing food choice is more carefully and clearly examined and concluded; (3) compared to studies from other field, the complex mechanisms and interactions between food perception (food-internal factor) and bio-physiological (personal-state) still need more investigation and the results should be integrated into the conceptual models of food choice; (4) while some factors affecting food choice could be universal (e.g., life course, (see [48]), large-scale and cross-cultural studies are needed to address factors influencing cultural-specific choices (see [123]). In addition, considering the obesogenic environment nowadays, despite the growing body of literature focusing on the role of cognitive function and food environment in food choice e.g., [124,125,126], we would like to emphasize the importance of understanding the complicated cognitive decision-making process (see [55]) and disentangling the interaction between cognitive functions and food environments, especially food-related information and physical environment (food-external factor) in order to develop effective intervention for helping individuals make better choice that is good for human health and the planet [127,128,129].

About the direction of influence among the factors in the conceptual models, fewer conceptual models are supported with empirical data [57,60,62,63,64,74,78,91,92,93,96,102,106,109].

Table S2 in the Supplementary Materials summarizes the directions of influence of factors affecting food choices based on publications with empirical data we included. We observed that factors proposed in theory of planned behavior (attitude, norms, and intention) and their effects are frequently examined with experimental settings and thus empirical data was obtained. Some other factors and the effects are also frequently investigated such as liking, food or nutritional knowledge, personal values, emotion, income, and sensory properties of food. Future studies should explore other factors and their effects on food choices.

In conclusion, the more specific factors the model addressed, the clearer indications of direction of influence could be made, supported by experimental data. We appreciate the multifactorial nature of individual food choice and the effort of including as many factors as possible in the models in providing a more intact and holistic view. However, the trade-off of expanding the models should be recognized too. In this case, interdisciplinary research is expected for constructing a holistic conceptual model of food choice supported by empirical data from studies in different fields (see [55,89]).

### 4.2. Implication of Factors Influencing Healthy and/or Sustainable Food Choice

In recent years, research has shed light on the factors affecting food choice towards healthier and more sustainable products. New conceptual models of food choice have been proposed to further depict how different factors essentially affect healthier and sustainable food choices. Through the literature review, the models were framed according to four types of choices: (1) healthier food choice [24,68,79,80]; (2) sustainable food choice [22,24,81,107,111]; (3) organic food [21,24,25]; and (4) fruit and/or vegetable [69,70]. 

For food-internal factors, the nutritional properties and the health value of the food items are especially important for the choice [68,69]. Health value [130] is an important index, including absence of contaminants [131]. For food-external factors, information based on nutrition facts, sustainability labels, and organic identity were included in the models as important drives [21,22,23,24,79,80,81]. Moreover, some studies pointed out that certification of origin and food miles, recycling packaging, as well as indications of local, traditional, ethic, and environmentally friendly products, can affect the food choice [130,131,132]. Social interaction and engagement in social institutions in social environment are important for making food decision, especially for fruits, vegetables, and organic food [21,23,70]. For physical environment, the availability of healthy or sustainable food products and the accessibility to nutrition environment, supermarket, or local stores are critical [23,80,130,133].

As personal-state factors, psychological components such as personality [23] as well as emotion [25,133] have impacts on healthier and organic food choice. Some other studies also mentioned motivation and intention as important factors contributing to the final food choice [23,134,135,136,137]. Cognitive factors such as belief, attitude, awareness, self-concept, and positive outcome expectation were addressed in determining food choice [21,22,23,24,25,68,69,70,79,80,81,107]. Finally, sociocultural context and familiarity based on cultural habits could affect food choices [69,70]. Income and economic situation are still a crucial determinant [69,79]. Food price heavily affects whether consumer will choose healthier and more sustainable food or not [69,70,130,131,132,137]. For making healthier and more sustainable food choice available, policy plays an essential role not only with healthy food eating policy but also with policies related to food supply chain, especially how food is produced, and environmental sustainability [35,88].

### 4.3. Future Research Directions

The present review provides an analytic framework of disentangling the complex and multifactorial nature of individual food choice with the aim of shifting towards healthier and more sustainable food systems. Recently, the European Commission has placed consumer food choice as one of the important targets for achieving a more healthy and sustainable EU food system. In 2017, over 950,000 deaths in EU (1/5 of population) were related to unhealthy diets. Thus, the “Farm to Fork” strategy [138], at the core of the European Green Deal, was launched in May 2020 which emphasized the importance of shifting towards healthier and sustainable diets in the EU by empowering citizens as consumers, to reverse the overweight and obesity trends, as well as to lower the environmental footprints. In addition, Outcome Report FOOD 2030 Pathways Workshop [139] indicated a pathway of research and innovation to develop innovative, healthy, sustainable and personalized nutrition solutions to reduce risk factors for Noncommunicable diseases, malnutrition, and micronutrient deficiencies. More importantly, consumers will be empowered to have a long-lasting, healthy, pleasurable, nutritional and sustainable diet tailored to individual parameters. It has been pointed out that there is a need for research to better understand the factors influencing consumer choice such as food environment, policies, gender, information, education, marketing, incentives, and lifestyles. 

In line with the goal set by the European Commission in the transformation of food systems, we confirmed that citizens as consumers—and their choices—should be placed at the very center of the problem of both human and planetary health, which calls for urgent investigation and solutions from academic field in providing opportunities and possibilities. Thus, it is crucial to understand individual food choice, factors affecting the choice, and possible interventions. Haddad, et al. [140] have proposed a new global research agenda for food with ten research priorities suggested. Among them, identify entry points for change, agree on what constitutes a healthy diet, make more data on diets widely available, tackle different forms of malnutrition simultaneously, study supply and demand, and identify the economic levers for change are closely related to addressing the important role of consumers’ choices in realizing healthier and more sustainable food systems. Lusk and McCluskey [141] further identified priority areas for future research, including dietary-related diseases and efficacy of policies designed to improve dietary choice, trust in the food system and acceptance of new food and farm technologies, environmental impacts of food consumption, changing consumer preferences, and food safety. Regarding data quality and availability, uncertainty in underlying biological and physical sciences, as well as applications of behavioral economic remain as challenges. In our opinion, future research examining factors influencing individual’ food choice should also take into account: (1) the need of multidisciplinary impulses and collaboration across research field such as sensory science, cognitive science, social science, as well as business studies; (2) the structural and systematic way (e.g., using the proposed framework as guiding map) of investigating the effect of food-choice related factors as well as the interactions among the factors; (3) the trade-off between the number of factors included in the framework and the capability to investigate and clearly explain the effect of s single factor and the effect of combining different factors; and (4) enriching and improving the framework with empirical observation or data based on a feedback mechanism.

### 4.4. Limitations

The present review has three main limitations. First, we focus on the three-level categorization of factors influencing food choice, namely food-related features, individual differences, and society-related features. However, recent research also pointed out the important roles in determining food choice, played by natural environment such as climate change [142,143,144,145], natural resources [146,147,148], as well as food production and supply chain [149]. Ideally, these factors should be also included into the conceptual framework. Second, the present study summarizes factors affecting food choice proposed in the conceptual models. The keywords we used for literature search may limit the inclusion of publications investigating a single or fewer factor(s) with experimental settings and empirical data. Thus, more meta-analyses are needed for understanding how different but specific factors contribute to food choice. Finally, this review does not include publications focusing on methodology used for understanding factors affecting food choices such as the development and validation of questionnaires (e.g., food choice questionnaire), which contain valuable information about the constructs of factors that are crucial for food choice decision. Thus, further examination with the inclusion of the results of factor analyses from food-choice-related questionnaires is recommended.

## Figures and Tables

**Table 1:** Factors influencing individual food choice included in our proposed framework.

Five Main Factors in Three Categories		Sub-Factors Under Five Main Factors
Food-related features	Food-internal factor	Sensory features (flavor, taste, smell, and texture) and perceptual features (color, portion size, nutrition and health value, and quality)
	Food-external factor	Information (nutritional labels, health claims, packaging, aesthetics, and ethics of production history, brand, advertisement)
		Social environment (intrapersonal factor and social norms from family, peers, and media including ethical concern, social context when food choice is made).
		Physical environment (availability and accessibility of food products, food retail environments, time).
Individual differences	Personal-state factor	Biological features (genetic factors, personal dietary patterns and metabolism, physical condition such as health).
		Physiological needs (hunger, appetite, and weight status)
		Psychological components (emotion, motivation, personality)
		Habits and experiences
	Cognitive factor	Knowledge and skills
		Attitude, liking and preference
		Anticipated consequences
		Personal identity (demographic features such as age, gender, ethnic identity, and education, and personal value and belief)
Society-related features	Sociocultural factor	Culture (norms and values)
		Economic variables (Income, socioeconomic status, and price)
		Political elements (Agricultural and food policy and regulations)

## Supplementary Materials

The following are available online at https://www.mdpi.com/2304-8158/9/12/1898/s1, Table S1: Factors included in the conceptual models of food choice. Table S2. Direction of influence of factors affecting food choices based on publications with empirical data.
