# Supplementary material for: Conceptual Models of Food Choice: Influential Factors Related to Foods, Individual Differences, and Society
Source: Foods. 2020 Dec 18;9(12):1898. doi: 10.3390/foods9121898 (PMC7766596; doi:10.3390/foods9121898)
Supplement: Supplementary file 1 [file foods-09-01898-s001.pdf]

Table S1. Factors included in the conceptual models of food choice.

Reference	Type of Publication	Keywords	Factors Included in the Model	Main Findings
Aertsens et al. (2009) [25]	Review	consumers, attitudes, organic foods, consumer behavior	Emotions, subjective norm, personal moral norm, perceived behavioral control, values, beliefs, attitude, experience, intention, perceived abilities and barriers, socio-demographic factors.	Relating attributes of organic food with more abstract values such as "security", "hedonism", "universalism", "benevolence", "stimulation", "self-direction" and "conformity" can positively influence attitudes towards organic food consumption. Subjective and personal norm and (perceived) behavioral control also influence consumption of organic food.
Asioli et al. (2017) [21]	Review	clean label, consumer preferences, food industry, review, drivers, trend, food products	Intrinsic product characteristics: Health, Sensory attribute, Nutritional properties.<br>Extrinsic product characteristics: Sustainability, Labeling and certification, Presence of health or nutritional claims, High price<br>Biological and Physiological factors: Age, Gender<br>Psychological factors: Health worries<br>Situational factors: Availability, Retail outlet<br>Sociocultural factors: Higher education, Participation in cultural activities, Personal values and ethical motivations, Trust in regulations and certification body, Additional information	'Health' is a major consumer motive. Moreover, intrinsic or extrinsic product characteristics and socio-cultural factors particularly influence the clean label trend.
Barreiro-Hurlé, Gracia, and de-Magistris (2010) [79]	Original Article	nutrition label use, nutrition knowledge, claims, facts panel, spain	Individual characteristics, consumer involvement with regards to food products, economic condition and time pressure, product information search, health awareness, habits, and status, lifestyles, nutrition knowledge, nutrition label use.	The use of nutrition information by consumers, no matter the fact panel or the claim labels, influence consumer choice of healthier food products. However, different types of consumers use the various types of labels considered.
Bisogni et al. (2005) [90]	Original Article	qualitative, biographical, life course, food choice, capacity, food management, cooking, food shopping	Life course events and experiences, circumstances (changing resources), standards (persistent and changing expectations), food management skills (double resources), food choice capacity.	A conceptual model of food choice capacity emerged, representing participants' confidence in meeting their standards for food and eating given their food management skills and circumstances. Standards (expectations for how participants felt they should eat) were based on life course events and experiences. Food management skills (mental and physical talents to keep food costs down and prepare meals) were sources of self-esteem. Changing circumstances (income, employment, social support, roles, health conditions) can be a challenge. Strong food management skills are related to high levels of food choice capacity,

				except in the case of extreme financial circumstances or the absence of strong standards.
Carfora, Caso, and Conner (2016) [91]	Original Article	fruit and vegetable, intake, theory of planned behavior, self-identity	Perceived behavior control, subjective norm, attitude, self-identity, past behavior, intention, future behavior.	Structural Equation Modelling indicated attitude, perceived behavior control, and self-identity to be significant predictors of intention. Intention, self-identity, and past behavior were direct predictors of behavior. There was an independent effect of self-identity as a healthy eater on both intentions and future behavior when controlling for Theory of Planned Behavior (TPB) variables and past behavior.
Coff, Korthals, and Barling (2008) [53]	Book chapter	n.a.	Information; Prestige; Culture and tradition; Social context, care for relatives; Identity; Price; Consumer perception of quality, taste and aesthetics; Availability, convenience; Health; Consumer concerns (ethics of production history); Voice.	Many different and opposing interests must be weighed against each other for making food choices. The priority of the interests may vary over time, depending on the situation, the mood of the consumer or the social contexts at the moment of shopping.
Connors et al. (2001) [54]	Original Article	demand, environment, food choice, health, sustainability.	Life course; Influences (Ideals, Personal factors, Resources, Social factors, Context); Personal food system (value negotiations, Managing relationships, Health, taste, cost, Convenience, Other); Strategies.	Within personal food systems, people managed the five main food-related values of taste, health, cost, time and social relationships, and other less prominent values of symbolism, ethics, variety, safety, waste and quality. The salience of these values varied among the participants as well as across the eating situations that confronted each participant. Participants used three main processes in their personal food systems: (1) categorizing foods and eating situations; (2) prioritizing conflicting values for specific eating situations; and (3) balancing prioritizations across personally defined time frames.
Contini et al. (2020) [92]	Original Article	structural equation modelling, Healthy food choices, Ready-to-eat foods, Convenience orientation, Lifestyles	Attitude, subjective norm, intention, cooking skills, health interest, market availability, time pressure, monetary resources, behavior.	A group of consumers considered plant-based convenience foods could improve their diet. For this cluster, the factors that significantly influence behavior are market availability, interest in healthy eating, and time pressure.
Costa-Font and Gil (2009) [109]	Original Article	genetically modified food, risks perceptions, benefit perceptions, structural equation modelling, Mediterranean Europe	Attitude towards science & technology, trust on experts and regulations, perceived benefits of genetic modified food (GMF), perceived risk of GMF, attitudes towards GMF, purchase intention of GMF.	Public attitudes toward GM food are formed from a reasoning mechanism based on trust in science and in public authorities, determining consumer's final purchasing decisions. Marked differences in the reasoning mechanism that lead to the acceptance of GM food in the three

				European countries indicating different food communication strategies to each culture.
Darnton and Evans (2013) [59]	Government publication (advice and guidance)	n.a.	Individual: Values, beliefs, attitudes, cost and benefits, emotions, agency, skills, habit.<br>Social: Roles and identity, taste, meanings, networks and relationships, norms, institutions, opinion leaders.<br>Material: Infrastructure, objects, time and schedules, rules and regulations, technologies.	This user guide introduces and explains the Individual, Social and Material contexts and the different factors that influence behaviors, with various examples that illustrate the applicability of ISM for successfully influencing behaviors. It is designed for policy makers and practitioners whose work ultimately aims at engaging people and influencing their behaviors in order to deliver improved outcomes.
de Boer, Hoogland, and Boersema (2007) [60]	Original Article	sustainability; food choices; values; motivational orientations; attitudes; meat	Personal values, personal and social characteristics, food choice motives, value-related attitudes, taste-related attitudes.	Most of the basic human values were related to the direction of the food choice motives. While universalism has impact on food choices favoring less meat or free-range meat. This impact was weak but robust and it was mediated by prevention-oriented food choice motives together with a high level of involvement in food and motive-congruent animal friendly attitudes.
Eertmans, Baeyens, and Van den Bergh (2001) [34]	Review	n.a.	Food-internal stimuli (Flavor), Food-external stimuli (Information, Social environment, Physical environment), Liking, Anticipated consequences, Ideational factors.	A hypothetical model of food choice including factors influencing eating behavior was proposed. Internal factors include sensory food aspects. External factors are information, the social context, and the physical environment. Processes such as mere exposure, Pavlovian conditioning and social learning shape the relationships between these factors, food liking and eating behavior. Liking for the sensory aspects of food is crucial for the development, maintenance and change of dietary patterns.
Farragher, Wang, and Worsley (2016) [93]	Original Article	vegetable consumption, personal values, demographics, personality, food knowledge, food mavenism, food involvement, survey, Australia	Age, gender, education, material status, number of children, conscientiousness, equality-universalism, food knowledge, food involvement, food mavenism.	The three types of vegetable consumption (salad vegetables, dinner vegetables, and green vegetables) and total servings per day were associated with different pathways. The mediating roles of food mavenism, food knowledge, food involvement and equality-universalist values may present opportunities for health promotion and the horticultural industry to increase population vegetable intake.

Fernqvist and Ekelund (2014) [61]	Review	consumer attitudes, quality perception, hedonic liking, extrinsic characteristics, credence cues	Product (Product characteristics): physical product, intrinsic characteristics, extrinsic characteristics.<br>Consumer quality perception process (signals/information): quality attributes, intrinsic quality cues, extrinsic quality cues, credence cues.<br>Consumer quality perception process (personal process): quality expectation influence by personal, contextual, and environmental factors (values/beliefs/attitudes; gender/age/lifestyle/other socio-demographic; situation/environment).<br>Consumer quality perception process (consumer quality perception): experienced quality.	Relevant literature concerning the effect of credence cues on consumers' hedonic liking of food was reviewed. A conceptual model based on a framework of consumers' quality perception process was applied, with seven main categories of credence characteristics being identified: health, organic food, origin, brand, production methods, ethics, and descriptive food names and ingredients.
Finch and Garnett (2016) [94]	Website resources	n.a.	Level 1: personal food preference ad tastes, family and local cultural norms, individual knowledge and skills, religious practices or other belief-driven norms.<br>Level 2: Food accessibility (storage and transport infrastructure, availability, etc.), food quality and safety, technology (cold storage infrastructure, transport, ICT, automation, packaging, etc.), social and wider cultural norms (mass media, education, structure of day, etc.), food prices (affordability).<br>Level 3: biophysical influences (environmental/climate change and resource constraints), agri-business and other corporate influences (supply chain developments, change in corporate power, ownership and structures, technological innovations, etc.), national and regional influences (agricultural policies, planning regulations, corporate governance, food legislation and labelling, dietary guidelines, public health campaigns, etc.), demographic influences (rising incomes, education, urbanization, etc.), civil society influences (awareness and concern around sustainability, health, animal welfare), Global influences (trade agreements, imports/exports, labor migration, environmental and phyto-sanitary agreements, etc.).	Levels of economic development, agricultural policy, pricing strategies, changes in how food is produced and distributed, marketing and media, values and aspirations, nutritional knowledge and access to information, and traditional attitudes to food and health, all influence consumption patterns. The role of policy is crucial by shaping the overarching social, infrastructural and economic influences on consumption and the extent to which health consequences are addressed.
Franchi (2012) [75]	Original Article	food choice, consumer behavior, culture, preferences, social representations	Axis of influences: parental models, media, brands, ties/resources, income, time available, instruction.<br>Axis of interpretations: experiences, relations, life courses, credence, values, identity.	This paper explored the complex mix of influences on food choice such as beliefs and identity, conditioned by social images that influence preferences, stressing that food choice is driven by identity. Taste involves beliefs and identity as well as perceptions, which has to be analyzed as a cultural and relational object.
Furst et al. (1996) [48]	Original Article	n.a.	Life course<br>Influences: social framework, food context.<br>Personal system: value negotiations (sensory perceptions, monetary considerations, convenience health and nutrition, managing relationship, quality), strategies.	People's life course experiences are major influences on food choice. Ideals, personal factors, resources, social contexts, and the food context affect the formation of personal systems for making food choices. In the personal system,

				value negotiations including sensory perceptions, monetary considerations, health and nutrition beliefs and concerns, convenience, social relationships, and quality of food choice decisions. Strategies involves in simplifying the food choice process developed over time.
Gains (1994) [71]	Book	n.a.	Food: taste, smell, texture, nutrients, image, packaging.<br>Consumer: habits, culture, personality, mood, physiology.<br>Context: time, place, who with, how, what with.	The interaction between the food itself, the consumer, and the context together influence the food choice behavior.
Glanz et al. (2005) [80]	Review	n.a.	Policy variables: government and industry policies.<br>Environmental variables: Community nutrition environment (type and location of food outlets such as stores and restaurants; accessibility such as hours of operation and drive-through), organizational nutrition environment (home, work, school, other), consumer nutrition environment (available healthy options, prices/promotion/placement, nutrition information), information environment (Media, advertising).<br>Individual variables: Sociodemographic (psychological factors, perceived nutrition environment).	a conceptual model based on an ecological model of health behavior was proposed, incorporating constructs found or hypothesized to be related to the healthy eating outcomes from the fields of public health, health psychology, consumer psychology, and urban planning. Food environments are shown as having two pathways of influence on eating patterns. Environmental effects can be moderated or mediated by demographic, psychosocial, or perceived environment variables. Environmental, social, and individual factors influence eating patterns, which in turn affect risk of many chronic diseases.
Grunert et al., (1996) [51]	Book chapter	n.a.	Before purchase: cost cues, perceived cost cues, perceived costs, extrinsic quality cues, perceived extrinsic quality cues, intention to buy, technical product specifications, intrinsic quality cues, perceived intrinsic quality cues, expected quality, expected purchase motive fulfilment.<br>After purchase: meal preparation, experience quality, experience purchase motive fulfilment, sensory characteristics, future purchase.	The Total Food Quality Model can serve as an overall framework for the analysis of consumers' food quality perception and its relation to buy and to the design of food product.
Grunert (2011) [22]	Original Article	sustainability, consumer behavior, hierarchy of effects, eco-labels	Eco-label, exposure, perception, understanding, inference-making, liking, motivation, awareness credibility.	Six barriers which may prevent consumers from using the information to make sustainable choices were discussed: exposure does not lead to perception, perception leads only to peripheral processing, consumers make 'wrong' inferences, eco-information is traded off against other criteria, lack of awareness and/or credibility, lack of motivation at time of choice.
Grunert, Hieke, and	Original Article	consumer behavior, food labelling, sustainability	Motivation: values, general concern about sustainability, product-specific concern about sustainability.	Generally, there is a low level of use of sustainability labels, no matter is measured as self-reported use of different types of

Wills (2014) [81]			Understanding: of the concept of sustainability, of sustainability labels.<br>Use of sustainability labels in food choice	information available on food labels or as use inferred from the results of a choice-based conjoint analysis. However, use is related to both motivation and understanding affected by demographic characteristics, human values and country differences.
Gutjar et al. (2015) [67]	Original Article	emotions, valence, arousal, Es Sense profile, food choice, liking	Sensory (Intrinsic) food information, Package (Extrinsic) food information, Emotional responses to foods (Valence, Arousal), Liking.	Food-evoked emotions may guide consumers' product choice behavior, adding predictive value to liking ratings. Liking and valence together had the strongest predictive value for product choice based on the product's taste. The combination of liking, valence and arousal had the strongest predictive value for package-based choice.
HLPE (2017) [76]	CFS HLPE documentation	n.a.	Food environment: food availability and physical access (proximity), economic access (affordability), promotion/advertising/information, food quality and safety.<br>Food supply chains: production system, storage and distribution, processing and packaging, retail and markets.<br>Drivers: biophysical and environmental drivers, innovation, technology and infrastructure drivers, political and economic drivers, socio-cultural drivers, demographic drivers.	The conceptual framework contains food systems, their drivers, actors and elements which are interlinked and in continual adaptive cycles of growth, restructuring and renewal. The central role of the food environment in facilitating nutritious, healthy and sustainable consumer food choices is stressed, while taking into account the impacts of agriculture and food systems on sustainability in economic, social and environmental dimensions.
Jabs, Devine, and Sobal (1998) [77]	Original Article	n.a.	Information, perceived threat of disease, animal connection, reasoning, life transition, incorporate into beliefs, physical aversion, further elimination.	Health vegetarians were motivated by a perceived threat of disease and the potential health benefits. Ethical vegetarians were motivated by moral considerations aligning dietary behaviors with beliefs and values about animal welfare. Information about the health and ethical impacts of vegetarian diets, physical aversions to animal-derived food, and life transitions, influences the decision of adopting a vegetarian diet.
Kang, Jun, and Arendt (2015) [68]	Original Article	value–attitude–behavior model, healthy foods, healthy eating, restaurants, expectations, health value	Health value, hedonic expectation, interest in healthy food, positive outcome expectation, healthy food choice intention.	At casual dining restaurants, health value was the key driver of customers' interest in healthy eating, aroused hedonic as well as positive outcome expectations, and thus enhancing the intentions to purchase healthy food items.
Hansen, Sørensenb, and Eriksenc (2018) [23]	Original Article	organic food behavior, organic food identity, personal values, consumer organic food motivations	Basic model: Environmental consciousness, health consciousness, social consciousness, organic food identity, intentional organic behavior.	Relationships between consumer motivations (health, environmental, and social consciousness), organic food identity, and organic food behavior were examined, under

			Personal values (moderating variables): Conservation, openness to change, self-enhancement, self-transcendence.<br>Control variables: Organic food involvement, social norm, perceived organic food price, gender, age, education, income.	the influence of different levels of personal values. Health consciousness has a higher positive influence on organic food identity. When openness to change is low, health consciousness has a positive effect on intentional organic food behavior through organic food identity, whereas social consciousness has a negative effect on intentional organic food behavior through organic food identity.
Kaufmann, Panni, and Orphanidou (2012) [82]	Original Article	pro-environmental consumer behavior, sustainability, green consumer behavior, green purchasing	Mediating variable (Demographic variables): Age, gender, income level, education level, ethnicity, occupation.<br>Independent variable: Environmental knowledge, altruism, environmental awareness, environmental concern and attitude, belief about product safety for use and availability of product information and product availability, perceived consumer effectiveness, collectivism, transparency/fairness on trade practices (customer care, product adulteration, unfair pricing black marketing, misleading advertising, deceptive packaging).	In the proposed integrated framework, eight important factors, as independent variables, have an impact on consumer green purchasing behavior. Demographic variables play a mediating role in the framework.
Kittler and Sucher (2004) [66]	Original Article	n.a.	Taste, variety, well-being, self-expression, convenience, cost.	An applied approach to multicultural competency through understanding the role of taste in food choices was provided, with proposed in Consumer Food Choice Model, including taste, cost, convenience, self-expression, well-being, and variety. The influence of taste on food choices should not be underestimated due to the physiological fondness for sweet, salty, and fatty flavors in the plentiful food environment which may lead to overeating and the conditions associated with obesity, including type 2 diabetes.
Hoek et al. (2017) [24]	Original Article	choice experiment, food choice, health, environment, sustainability, price, consumer	Standard food product: Price, logo, label.<br>Consumer: Attitudes, knowledge, product familiarity and liking, sociodemographic.<br>Healthy and sustainable food product: Price, logo, label.	The similarity to the standard products for their sensory properties, convenience, product liking and familiarity influence point-of-purchase actions of choosing healthy and sustainable foods. Decreased price (subsidy) for the healthy and sustainable alternatives showed bigger effect on shifting choices than did a logo and/or label.
Köster (2009) [55]	Original Article	sensory consumer research, food-related behavior, interdisciplinarity, intuitive reasoning, non-conscious decision making, situational	Intrinsic product characteristics: Appearance, Interaction Taste Smell, Texture, Trigeminal; Complexity Adaptation, Dynamic contrast; Irritation, Boredom, Aversion.<br>Extrinsic product characteristics: Claims/brand label packaging, Integrity Sustainability, Risk perception.	The contribution of intuitive reasoning and unconsciousness to the decision making do not seem to be included in sensory and consumer research. Past behavior, habit and hedonic appreciation are usually better predictors of

		analysis, interactive integration of behavior determinants.	Biological and Physiological factors: Oro-gastro-intestinal physiology; Age/Gender, Physical condition, Sensory acuity; Genetic factors, Immune system, Brain imaging.<br>Psychological factors: Cognition, Emotion/motivation, Decision Making; Memory, Previous experiences, Learning; Personality traits, Neophobia<br>Situational factors: Time, Social surroundings, Physical surroundings; Coping, Assimilation, Habituation; Intentionally, Signification, Attribution.<br>Socio cultural factors: Cultural economical Influence; Trust in industry and government; Changing beliefs, norms, habits, attitudes.	actual food choice behavior than psychological constructs like attitudes and intentions.
Krebs-Smith and Kantor (2001) [69]	Supplement	fruits, vegetables, diet surveys, dietary assessment, food supply	Environmental factors, agricultural factors, economic factors, policy considerations, income and price, sociocultural factors, demographic factors, educational factors, environmental factors, characteristics of food, food preferences, cognition attitudes, physiological factors.	The 2000 edition of Nutrition and Your Health: Dietary Guidelines for Americans is the first to include a specific recommendation for fruits and vegetables. Fruit and vegetable consumption appears to be rising, but this slight increase might be only an artifact of shifts in the population demographics. Low-income households in poor central cities and sparsely populated rural areas often have less access to food stores and face higher prices for food such as fruits and vegetables, compared with other households.
Kumar, Manrai, and Manrai (2017) [107]	Original Article	environmentally sustainable products, collectivistic culture, moderated mediation	Environmental knowledge, attitude, subjective norm, purchase intention.	The attitude towards environmentally sustainable products mediates the relationship between environmental knowledge and purchase intention. A collectivistic culture instead of the subjective norm, is related to the purchase intention.
Kushwah, Dhir, and Sagar (2019) [62]	Original Article	buyer and non-buyer, environmental concerns, ethical consumption, organic food, theory of consumption values	Functional value (quality), functional value (price), social value, emotional value, conditional value, epistemic value, environmental concern, ethical consumption intention, choice behavior.	Significant association between social, emotional, and epistemic values and ethical consumption intentions was found. Epistemic value influenced both ethical consumption and choice behavior. Consumers with varying levels of environmental concerns showed the difference in associations of epistemic and price-related functional values and ethical consumption intentions.
Kushwah, Dhir, and Sagar (2019) [78]	Original Article	consumer resistance, organic food, barriers, innovation resistance theory (IRT), sustainable consumption	Image barrier, value barrier, risk barrier, purchase intention, ethical consumption intention, buying involvement/environmental concern.	Value barrier negatively associated with purchase intentions and ethical consumption intentions. Ethical consumption and purchase intention were found to have a direct influence

				on choice behavior. Relationship between ethical consumption intention and choice behavior is mediated by purchase intention.
Lartey, Hemrich, and Amoroso (2016) [32]	FAO documentation	n.a.	Food supply subsystems: Food storage, transport, and trade; food transformation; food retail and provisioning; agricultural production.<br>Food environment: Food labelling, food promotion, food price, physical access to food, nutrient quality and taste of available food.<br>Consumer: Purchasing power	A conceptual framework explained related links between food systems, food environments, consumer choices and diet. Four food supply subsystems comprise the entire "farm to fork" food chain. These subsystems influence the food environments in which people make their dietary choices.
Mai et al. (2015) [95]	Original Article	health, food, nutrition self-efficacy, implicit association test, mediated moderation.	Mediated moderation: nutrition self-efficacy.<br>Control variables: social-cognitive predictors of health behavior change (outcome expectancy, risk perception), socio-demographics/psychographics (sex, hunger, diet, education, positive feelings, impulsiveness, household size).<br>Implicit food associations<br>Intention to adopt a healthy diet	Consumers with a low ability to adopt a healthy diet behave according to their self-efficacy if implicit associations are negatively connoted. A mediated moderation model showed that the interaction effect exerts its influence via intentions to adopt a healthy diet.
Marreiros and Ness (2009) [56]	Working Paper	consumer behavior, food, decision-making, perception, quality.	Stage 1: Environmental influences (culture, social class, personal influences, family, situation), Individual differences (consumer resources, motivation and involvement, knowledge, attitudes, personality, values, and lifestyle).<br>Stage 2: Need recognition, search/internal search/external search, memory, pre-purchase/post-purchase alternatives evaluation, satisfaction/dissatisfaction.<br>Stage 3: Product specifications, perceived intrinsic quality cues, perceived extrinsic quality cues, expected quality, perceived cost cues, perceived cost, expected value, experience quality.	A two-component conceptual framework for analyzing consumer decision-making towards fresh food was proposed, incorporating the main features of the Engel-Blackwell-Miniard Model of Engel et al. (1995) regarding consumers decision-making, and the main constructs of the Total Food Quality model from Grunert (1997), concerning consumers quality evaluation. Additional constructs and relationships between them, proposed by Zeithaml (1988) and by the authors of the present paper, were added.
Mørk et al. (2017) [96]	Original Article	policy acceptance, organic food consumption, institutional catering, spillover	Values, attitude to environment, personal norms, social norms, purchase of organic produce, attitude to procurement of organic produce in public kitchen, persona; relevance.	Personal norms regarding the use of organic food affect attitudes toward the use of organic produce in institutional settings, which is partly mediated by own purchase of organic products. Collectivistic values and individualistic values affect personal and social norms on using organic product, partly mediated by attitude to the environment.
Olsen et al. (2012) [63]	Original Article	convenience food, ready-to-heat meals, likelihood of buying, at-home testing, liking, health orientation	Appearance, flavor, texture, odor, overall liking, health orientation, gender, age, education, likelihood of buying.	The likelihood of buying healthy convenience food is affected by overall liking of the meal, which is affected by liking of sensory specific product attributes like appearance, flavor, and odor. Effects of socio-demographic factors were found to be product dependent.

Oostenbach et al. (2019) [57]	Review	nutrition claims, influence, food choices, energy intake, overweight, obesity	Nutrition claim, weight status, nutrition knowledge, expected tastiness of product perceived nutrition characteristics of product, perceived appropriate portion size, motivation to adopt a healthy diet, emotions, experienced tastiness of product.	Nutrition claims have effects in (1) influencing the knowledge of consumers with respect to perceived healthfulness of products, as well as expected and experienced tastiness of food products; (2) making the appropriate portion size appear to be larger and lead to an underestimation of the energy content of food products; (3) influencing food purchase intentions, moderated by the perceived healthfulness of the relevant food products and the health consciousness of individuals; (4) having an impact on food purchases by influencing 'consumption guilt' (i.e., feeling of guilt associated with eating), as well as on increased consumption, moderated by the weight status of individuals.
Perry et al. (2017) [97]	Review	scoping review, food literacy, food skills, attributes	Intrinsic: Food skills (food techniques, food skills across the lifespan), food and nutrition knowledge (food knowledge, nutrition knowledge, food language, nutrition language), self-efficacy and confidence (nutrition literacy, food self-efficacy, cooking self-efficacy, food attitude).<br>Extrinsic: ecologic (socio-cultural influences and eating practices, food and other systems, infrastructure and population-level determinants).	Fifteen identified food literacy attributes were organized into five categories. Food and Nutrition Knowledge informs decisions about intake and distinguishing between 'healthy' and 'unhealthy' foods. Food Skills focuses on techniques of food purchasing, preparation, handling, and storage. Self-Efficacy and Confidence represent one's capacity to perform successfully in specific situations. Ecologic refers to beyond self and the interaction of macro- and microsystems with food decisions and behaviors. Food Decisions reflects the application of knowledge, information and skills to make food choices.
Pollard, Kirk, and Cade (2002) [70]	Review	food choice, fruit, vegetables, health behavior	Availability: Shopping facilities, eating out.<br>Monetary cost<br>Time constraints: Shopping, preparation and consumption, perishable nature of foods.<br>Sensory appeal: Taste, texture, smell.<br>Familiarity: Habits, cultures and traditions, food neophobia.<br>Social interactions: Social desirability, food ideology.<br>Personal ideology: political belief, trade policies.<br>Media and advertising<br>Health: knowledge, optimistic bias.	Factors affecting food choice decisions of adults in relation to fruit and vegetable consumption were investigated and a suggested framework of food choice was proposed. Factors covered include sensory appeal, familiarity and habit, social interactions, cost, availability, time constraints, personal ideology, media and advertising and health.
Randall and Sanjur (1981) [41]	Original Article	vegetable, preference, food frequency, consumption, dislike, selectivity,	Characteristics of the individual: age, sex, education, income, nutrition knowledge, cooking skills and creativity, attitudes to health and the role of food to it.	The conceptual model with characteristics of the food, the individual, and the environment, was proposed to examine the relationship between

		conceptualization, sociocultural correlates	Characteristics of the food: taste, appearance, texture, cost, food type, method of preparation, form, seasoning, food combinations. Characteristics of the environment: season, employment, mobility, degree of urbanization, size of household, stage of family. Food preference Food consumption	food preference and consumption. Food preference seems to function as a limit on the available food supply for individuals, supported by weaker relationship between a like for and consumption compared to that between a dislike and non-use.
Ricci, Banterle, and Stranieri (2018) [111]	Original Article	consumer trust, convenience food, eco-friendly attributes, theory of planned behavior	Institutional trust, attitudes, perceived availability, food shopping habits, concern towards agricultural practices, intention to buy.	The conceptual framework was built on and extended the theory of planned behaviour. Consumer trust positively affects attitudes towards the purchase of convenience food with eco-friendly attributes while negatively affects consumer concerns around agricultural practices in relation to environmental and health impacts.
Rodríguez-Entrena and Salazar-Ordóñez (2013) [110]	Original Article	GM food, consumers' acceptance, consumers' literacy fields, Partial Least Squares	Attitude to GM technology, trust in institutions, knowledge, perceived benefits from GM food, perceived risk from GM food, GM purchase intention.	Perceived benefits and risks influence behavioral intentions towards GM food, while the attitude to GM technology being the main driver of consumers' beliefs about risks and benefits. Behavioral intentions display some differences between the scientific–technical- and social–humanistic-related consumers, driven by trust in institutions and knowledge.
Rose et al. (2010) [87]	Symposium paper	n.a.	Neighborhood food access (food store placement, in-store price in-store characteristics such as number of varieties, shelf space, placement), promotional effect, social acceptability, age/race-ethnicity/education, food cost (travel cost and car ownership), taste and preferences, income.	Shelf space availability of energy-dense snack foods is positively associated with BMI after controlling for individual socioeconomic characteristics.
Rosenfeld and Burrow (2017) [83]	Original Article	vegetarianism, veganism, identity, food choice, plant-based	Externalized Dimensions: Dietary pattern label, strictness. Internalized Dimensions: Salience, centrality, regard, motivation. Contextual Dimensions: Timing, duration, historical embeddedness. Timeline context: historical, sociocultural, lifespan.	The Unified Model of Vegetarian Identity was proposed, including ten dimensions organized into three levels (contextual, internalized, and externalized), capturing individual's self-concept. Contextual dimensions situate vegetarianism within contexts; internalized dimensions outline self-evaluations; and externalized dimensions describe enactments of identity through behavior.
Shepherd (1999) [49]	Original Article	food choice, social psychological attitudes, optimistic bias	Food: Physical and chemical properties, nutrient content. Physiological effects (satiety, hunger, thirst, appetite). Person: perception of sensory attributes (appearance, aroma, taste, texture). Psychological factors (personality, experience, mood, beliefs). Economic and social: price availability, brand, social and cultural.	Measuring moral concern for the choice of genetically modified foods and for foods to be eaten by others, is important. It has been found to be difficult to effect dietary change because of (1) optimistic bias, where individuals believe themselves to be at less risk from various

			Attitudes: to sensory properties, health and nutrition, price and value.	hazards than the average person. This effect has been demonstrated for nutritional risks, and this might lead individuals to take less note of health education messages. And (2) individuals can be ambivalent about food and about healthy eating.
Snoek et al. (2018) [98]	Review	research infrastructures, public health, roadmap, governance, policy, nutrition	Determinants of dietary behavior: psychosocial, contextual aspects, food and policy environment. Intake of food and nutrients: dietary assessment, food composition, analytical food chemistry. Status and functional markers: biomarkers and mechanisms, metabolic functioning. Health and disease risk of food and nutrients: infectious and chronic disease risk.	In the EuroDISH project, existing Research infrastructures in the food and health area in Europe are mapped with a proposed DISH model, describing and structuring the research area: Determinants of food choice, Intake of foods and nutrients, Status and functional markers of nutritional health, and Health and disease risk.
Sobal and Bisogni (2009) [47]	Original Article	food, eating, choice, decisions, construction, theories	Life course; Influences (Ideals, Personal factors, Resources, Social factors, Context); Personal food system (value negotiations, Managing relationships, Health, taste, cost, Convenience, Other); Strategies.	A food choice process model was developed based on constructionist social definition perspectives, including (1) life course events and experiences that establish a food choice trajectory through transitions, turning points, timing, and contexts; (2) influences on food choices that include cultural ideals, personal factors, resources, social factors, and present contexts; and (3) a personal system that develops food choice values, negotiates and balances values, classifies foods and situations, and forms/revises food choice strategies, scripts, and routines. Food choice decisions leading to food behaviors were affected by dynamic interaction of some parts of the model.
Sobal, Khan, and Bisogni (1998) [52]	Review	model, food, nutrition, health, agriculture, diet, systems	Social environment, utility, meaning, satisfaction, policy and institutions, capital, skills, knowledge, bio-physical environment, byproducts, waste, bio-diversity, land, air, water, energy.	Models of food chains, food cycles, food webs and food context were identified. An integrated model developed here included three subsystems (producer, consumer, nutrition) and nine stages (production, processing, distribution, acquisition, preparation, consumption, digestion, transport, metabolism), considering the processes and transformations that occur within the system and relationships between the system and other systems in the biophysical and social environments.
Steenkamp (1993) [50]	Review	n.a.	Properties of the food: Physical, chemical, nutrient content. Person-related factors: biological, psychological, personality. Environment: socio-cultural, economic, marketing.	A taxonomy of determinants of food consumption behavior was developed. The determinants were categorized as related to the

				food, the person, and the environment, with the highlight of the role of factors as diverse as age, satiety capacity of foods, and cultural taboos.
Story et al. (2008) [88]	Review	ecological framework, environmental and policy change, healthy eating environments	Individual factors (personal): Cognitions (attitudes, preferences, knowledge, values), skill and behaviors, lifestyle, biological (genes, gender, age), demographics (income, race/ethnicity). Mediators: outcome expectations, motivations, self-efficacy, behavioral capability.<br>Social environment (networks): Family, friends, peers. Mediators: role modeling, social support, social norms.<br>Physical environments (settings): Home, work sites, school, child care, neighborhoods and communities, restaurant and fast food outlets, convenience and corner stores. Mediators: access, availability, barriers, opportunities.<br>Macro-level environments (sectors): Societal and cultural norms and values, Food and beverage industry, Food marketing and media, Food and agriculture policies, Economic systems, Food production and distribution systems, Government and political structures and policies, Food assistance programs, Health care systems, Land use and transportation. Mediators: practices, legislative regulatory, or policy actions.	An ecological framework for conceptualizing the food environments and conditions that influence food choices was proposed, with an emphasis on current knowledge regarding the home, childcare,<br>school, work site, retail store, and restaurant settings. Disparities in food access for low-income and minority groups and macrolevel issues were also reviewed.
Stranieri, Ricci, and Banterle (2017) [102]	Original Article	consumer preferences, sustainability, convenience food, integrated pest management, environmentally-friendly attributes	Attitude towards integrated pest management minimally processed vegetables (IPM MP veg.), perceived availability of IPM MP veg., food chopping habits, agricultural practice concern, intention to purchase IPM MP veg., individual characteristics, food-related environmental behavior/	The purchase of healthy convenience food products had positive relations with consumer food shopping habits, food-related environmental behavior, gender, income, and knowledge. There was a negative relation with agricultural practices concern, suggesting that the most concerned consumers may prefer other more stringent environmental certifications.
Symmank et al. (2017) [89]	Review	food decision making, food choice, predictors, interdisciplinary, mapping review, consumer behavior	Individual: Biological, demographical, psychological, situational.<br>Environment: Product, micro, meso/macro.<br>Interpersonal: Social and Cultural.<br>Policy: Industry and government.	Systematic interdisciplinary mapping (SIM) was used to identify predictors of food decision making were categorized in line with the recently proposed DONE (Determinants of Nutrition and Eating behavior) framework. Results showed that most of the research emphasizes biological, psychological, and product-related predictors, whereas policy-related influences on food choice are scarcely considered.
Turner et al. (2018) [58]	Original Article	food environments, low- and middle-income countries, food security, food acquisition,	External domain: Availability (presence of food sources or products), prices (monetary value of food products), vendor and product properties (typology, opening hours, service, food quality, composition, safety, level of processing, shelf-life,	The socio-ecological theory driven conceptual framework aligned with methods and metrics by mapping geospatial and observational approaches to personal and external food

		double burden of malnutrition, non-communicable diseases	packaging), market and regulation (promotional information, branding, advertising, sponsorship, labelling, policies). Personal domain: Accessibility (physical distance, time, space and place, individual activity spaces, daily mobility, mode of transport), affordability (purchasing power), convenience (relative time and effort of preparing, cooking and consuming food product, time allocation), desirability (preferences, acceptability, tastes, desires, attitudes, culture knowledge and skills).	environment domains responds to the identified gap in linking food environment theory and concepts with methods and metrics. The conceptual framework depicts the food environment as the interface within the wider food system. Key dimensions are mapped to external and personal domains. Interactions between these domains and dimensions shape people's food acquisition and consumption.
van Buul et al. (2017) [64]	Original Article	food choice, MouselabWeb, process tracking, nutrition literacy, health action process approach, BOP nutrition information	Self-efficacy, outcome expectancies, risk perception, demographic factors, intention, planning, nutrition literacy and taste preference, quantity of nutritional information considered, healthiness of substitutive food choices.	For consumers who intended to eat healthy, the quantity of obtained nutrition information significantly associated with the percentage of healthy food choices made. Moreover, the level of correct answers in a nutrition literacy test, as well as taste preferences, significantly predicted the percentage of healthier choices.
Zeithaml (1988) [65]	Original Article	n.a.	Lower-level attributes: Intrinsic attribute, extrinsic attribute, objective price. Perception of lower-level attributes: Perceived monetary price, perceived nonmonetary price, perceived sacrifice. Higher-level attributes: Perceived quality, perceived value, high-level abstraction.	Evidence from previous studies and results from an exploratory investigation were combined in a conceptual model that defines and relates price, perceived quality, and perceived value.

Table S2. Direction of influence of factors affecting food choices based on publications with empirical data.

Reference	Significant Positive Effect	Significant Negative Effect	No Effect
Carfora, Caso, and Conner (2016) [91]	Perceived behavioral control, attitude, and self-identity positively predict the intention of fruit and vegetable intake. Past behavior and self-identity ($p < .001$) as well as Intention positively predicts future behavior of fruit and vegetable intake.	n.a.	Subjective norm and past behavior do not predict intention of fruit and vegetable intake
Contini et al. (2020) [92]	Attitude, subjective norm, health interest, and market availability have positive impacts on Intention of plant-based convenience foods consumption. Intention and time pressure as well as market availability have positive impacts on behavior of plant-based convenience foods consumption.	Cooking skills have negative impacts on Intention.	Monetary resources have no impact on either intention or behavior.
Costa-Font and Gil (2009) [109]	Perceived benefits of GMF has a positive impact on attitude towards GMF. Attitude towards GMF has a positive impact on purchase intention of GMF.	Perceived risk of GMF has a negative impact on perceived benefits of GMF. Perceived risk of GMF has a negative impact on attitude towards GMF.	n.a.
de Boer, Hoogland, and	Universalism has a positive impact on food choices for favoring less meat or free-range meat. This impact was mediated by prevention-oriented food choice motives together with a high	n.a.	n.a.

Boersema (2007) [60]	level of involvement in food and motive-congruent animal friendly attitudes.		
Farragher, Wang, and Worsley (2016) [93]	Food knowledge is positively associated with age, female gender, education, equality-universalism, as well as the total vegetables consumption per day.<br>Food involvement is positively associated to equality-universalism, age, food knowledge, food mavenism, as well as the total vegetables consumption per day.<br>Food mavenism is positively associated with female gender, being married or cohabiting, education, equality-universalism, as well as the total vegetables consumption per day.	Food knowledge and food involvement are negatively related to conscientiousness.<br>Food mavenism is negatively related to age.	n.a.
Gutjar et al. (2015) [67]	Actual food choice based on the products' sensory properties was best predicted by emotional responses (valence) and liking combined.<br>In the package choice context, food choice based on the products' extrinsic properties (package) was best predicted by two emotion dimensions (valence and arousal) and liking ratings.<br>Information on food product emotional profiles adds predictive value over liking for actual food choice behavior displayed by consumers.	n.a.	n.a.
Kushwah, Dhir, and Sagar (2019) [62]	Emotional value and epistemic value have a positive influence on ethical consumption intentions.<br>Epistemic value has positive influence on choice behavior.	Social values have a negative impact on ethical consumption intention.<br>Conditional value has a negative impact on choice behavior.	Functional value-quality, functional value-price, and conditional value have no significant impacts on ethical consumption intention.<br>Functional value-quality, functional value-price, social value, and emotional value have no significant impacts on choice behavior.
Mørk et al. (2017) [96]	Attitude to procurement of organic produce in public kitchens is strongly influenced by a personal norm to eat organic products, partly mediated by own purchase of organic produce.<br>Personal norms to consume organic products are affected by attitude to the environment and by social norms to consume organic products, which in turn are affected by scores on the universalism value (collectivistic) and to a lesser extent by the achievement value (individualistic value).<br>Universalism (collectivistic value), achievement (individualistic value), attitude to the environment, personal norms, and social norms all have significant indirect effects on attitude towards the procurement of organic produce in public kitchens.	n.a.	The effect of personal relevance is not significant.<br>The impact of personal norms and of purchase of organic produce on attitude to procurement of organic produce in public kitchens are not statistically significantly different, indicating no moderator effect of personal relevance.
Olsen et al. (2012) [63]	The main driver of likelihood of buying is overall liking of the meal, influenced by flavor, appearance, texture, and odor.<br>Flavor is the strongest driver of overall liking for the meals, stronger than the effect from liking of odor, appearance and texture.	n.a.	n.a.

	Gender, education, age, and health orientation have an effect for the likelihood of buying the salmon meal.		
Rodríguez-Entrena and Salazar-Ordóñez (2013) [110]	A positive attitude to GM technology increases the perceived benefits of GM food.<br>Trust in institutions positively influences the perceived benefits of GM food for socially–humanistically literate consumers.<br>For social–humanistic literacy consumers, the perceived benefits increase when there is a high level of knowledge<br>Perceived risks increase with a high level of knowledge for the scientific–technical literacy consumers.<br>Perceived benefits have a positive impact on GM food purchase intentions.	A negative attitude to GM technology increases the perceived risks for both socially–humanistically and scientifically–technically literate consumers.<br>Trust in institutions decreases the perceived risks.<br>Perceived risks have a negative impact on GM food purchase intentions	For scientific–technical literacy consumers, level of knowledge has no significant influence on perceived benefits.<br>For the social–humanistic literacy consumer, level of knowledge has no significant influence on perceived risks.
Stranieri, Ricci, and Banterle (2017) [102]	The likelihood of purchasing integrated-pest-management MP vegetables (behavior) can be positively predicted by the intention of performing such behavior.<br>The intention is positively guided by the evaluation of the behavioral outcome (attitude) related to the behavior.<br>The perceived ability to perform the behavior is positively and significantly associated with the intention to perform the behavior.<br>The positive association of food shopping habits reveals that consumer habitual actions during food purchase, like the search of labelled information, have a strong influence on the intention to buy convenience food like MP vegetables.<br>Knowledge regarding environmental attributes of MP vegetables is positively and significantly associated with the purchasing of IPM products.<br>Gender and income are significant and positively associated with the behavior.<br>The frequency of purchase of MP vegetables is also associated with the purchase of IPM.<br>Consumer food related environmental behavior has a positive effect on the choice of IPM MP vegetable	A strong negative relationship between the intention to buy IPM MP vegetables and the concern for the impact of agricultural practices.	n.a.
van Buul et al. (2017) [64]	In the full model, taste preference, nutrition literacy, and number of attributes considered all positively contributed as associative factors of the percentage of healthy food choices made.<br>Intention to eat healthy positively contribute to the percentage of healthy food choices made, but not in the full model	Self-efficacy negatively contributes to the percentage of healthy food choices made, but not in the full model.	Gender, BMI, age, action planning, coping planning, time of attributes considered are not significantly influencing the percentage of healthy food choices made.
